# Image-guided interstitial brachytherapy for recurrent cervical cancer after radiotherapy: A single institution experience

**DOI:** 10.3389/fonc.2022.943703

**Published:** 2022-07-19

**Authors:** Xiaojun Ren, Yingli Fu, Zhongshan Liu, Xia Lin, Ling Qiu, Yunfeng Li, Hanyang Li, Yuqi Bai, Tiejun Wang

**Affiliations:** ^1^ Department of Radiation Oncology, The Second Hospital of Jilin University, Changchun, China; ^2^ Department of Clinical Epidemiology, The First Hospital of Jilin University, Changchun, China

**Keywords:** reirradiation (re-RT), recurrence, cervical cancer, brachytherapy, interstitial

## Abstract

**Purpose:**

The aim of this study is to evaluate the efficacy and toxicity of image-guided high-dose rate (HDR) interstitial brachytherapy (ISBT) for the reirradiation of cervical cancer within a previously irradiated area.

**Methods and materials:**

Twenty-three consecutive patients with cervical cancer were reirradiated with curative intent using brachytherapy (BT) with or without external beam irradiation. The median biologically equivalent dose in 2-Gy fractions (EQD2) for reirradiation was 64.0 Gy (range: 31.3–95.1 Gy), and the median cumulative EQD2 (for primary treatment and reirradiation) was 152.4 Gy (range: 97.8–200.9 Gy). The average clinical target volume was 82.9 cm^3^ (range: 26.9–208.3 cm^3^), and the median treatment-free interval (TFI) was 13 months (range: 3–93 months).

**Results:**

The median follow-up time was 19 months (range: 2–59 months). The complete response rate after reirradiation was 56.5%. The 1-, 2- 3-, and 4-year post-relapse survival (PRS) rates were 65.2%, 43.5%, 33.8%, and 27.1%, respectively. The median reirradiation EQD2 D2cc of rectum and bladder was 39.5 Gy (range = 14.6–96.2 Gy) and 52.1 Gy (range = 29.1–114.2 Gy). The median cumulative EQD2 D2cc of rectum and bladder was 115.0 Gy (range = 84.4–189.3 Gy) and 130.5 Gy (range = 95.5–173.5 Gy). During follow-up, nine (39.1%) patients had experienced grade 3 or 4 late toxicities. Grade ≥3 rectal toxicity occurred in three patients (13.0%). Grade ≥3 urinary toxicity occurred in five patients (21.7%). One patient (4.3%) had both grade ≥3 urinary and rectal toxicity. Tumor volume, TFI, tumor invasion organ number, and local control were significant prognostic factors adversely affecting OS.

**Conclusions:**

For recurrent cervical cancer after radiotherapy, reirradiation of HDR-ISBT is feasible, even if the local tumor invasion is large, with a good chance of survival and acceptable side effects.

## Introduction

Cervical cancer is the fourth most common malignancy and the fourth leading cancer-related death in women worldwide (1). The recurrence rates of patients with IIA, IIB, IIIA, IIIB, IVA, and IVB stage cervical cancer were 21.2%, 27.8%, 40.9%, 44.7%, 64.3%, and 73.6%, respectively (2). A previous study reported that the recurrence rates of patients with stage IB, IIA, and IIB cervical cancer treated only by radiotherapy were 10%, 17%, and 23%, and the rates of patients treated by radiotherapy plus surgery respectively were 14%, 20%, and 29%, respectively (3). The 5-year survival rate for patients with cervical cancer who relapsed after radical surgery or radiotherapy was only 3.2%~13% (4, 5). The treatment of recurrent cervical cancer is challenging and mainly depends on the previous treatment and the site and extent of recurrence (2).

The retreatment of recurrent cervical cancer that develops in a previously irradiated field is a complex challenge for gynecologic oncologists (6). Surgery, which exhibits 5-year overall survival rates higher than 30%, could be a curative treatment option for patients who meet strict indications. However, surgery has been employed carefully because of a high rate of complications and positive surgical margins (7–10). In recent years, with the progress of radiotherapy technology and the development of computed tomography (CT)/magnetic resonance imaging (MRI)–guided brachytherapy (BT), the reirradiation of recurrent cervical cancer after radiotherapy has achieved a good curative effect, and the incidence of serious adverse reactions is low (11–15). Moreover, reirradiation can preserve the structure and function of the organ and thus improve the quality of life of the patients. Hence, reirradiation could be another potentially curative treatment option for recurrent cervical cancer after radiotherapy. However, there are few reports of reirradiation in patients with cervical cancer, and all of them are small sample studies. In addition, there are no recommendations in the literature for the reirradiation dose of interstitial brachytherapy (ISBT), either as a treatment alone or in combination with external beam radiation therapy (EBRT). This retrospective study aimed to evaluate the effectiveness and toxicity of high-dose rate (HDR)–ISBT as retreatment to develop clinical practice guidelines.

## Materials and methods

### Patients

A review of the database in the department of radiotherapy in the Second Hospital of Jilin University identified 23 patients who received HDR-BT reirradiation between November 2015 and August 2020 for a local recurrence (LR) occur within a previously treated volume, following radical or adjuvant RT for cervical cancer. Fifteen patients were not suitable for pelvic exenteration, and the others refused surgery for fear of surgical trauma. Twelve patients had LR identified by pathological examination, and the rest had LR confirmed by pelvic MRI and positron emission tomography (PET)/CT. Twelve patients were initially treated at other hospitals, and detailed doses of organs at risk (OARs) could not be obtained. When brachytherapy is used, radiation doses to the rectum and bladder should be limited to 65% and 75% of the tumor dose, respectively (16). The limit of the minimum dose delivered to 2 cm^3^ (D2cc) rectum and sigmoid colon was 70–75 Gy, and that of the bladder was 90 Gy (17). For patients who were unable to obtain an accurate dose from the bladder and rectum during primary brachytherapy, we uniformly calculated the D2cc at 70% of the prescribed dose.

### External beam radiation therapy

During reirradiation, the tumor location of some patients was too far from the vulva, leading to the use of external radiation therapy when the metal needles could not reach. Conformal EBRT was delivered to the tumors with a 0.7-cm margin using high-energy 6-MV photons with 1.8- to 2-Gy fractions to total doses of 30.0–50.4 Gy. The local tumor is the target of EBRT. The medical linear accelerator that we used is Varian Varianix-4702.The radiotherapy positioning system was Varian Eclipse 13.5. One hundred percent of the prescribed dose of EBRT was considered while calculating doses delivered to the tumor and the OARs.

### High-dose rate interstitial brachytherapy

**Figure 1:**
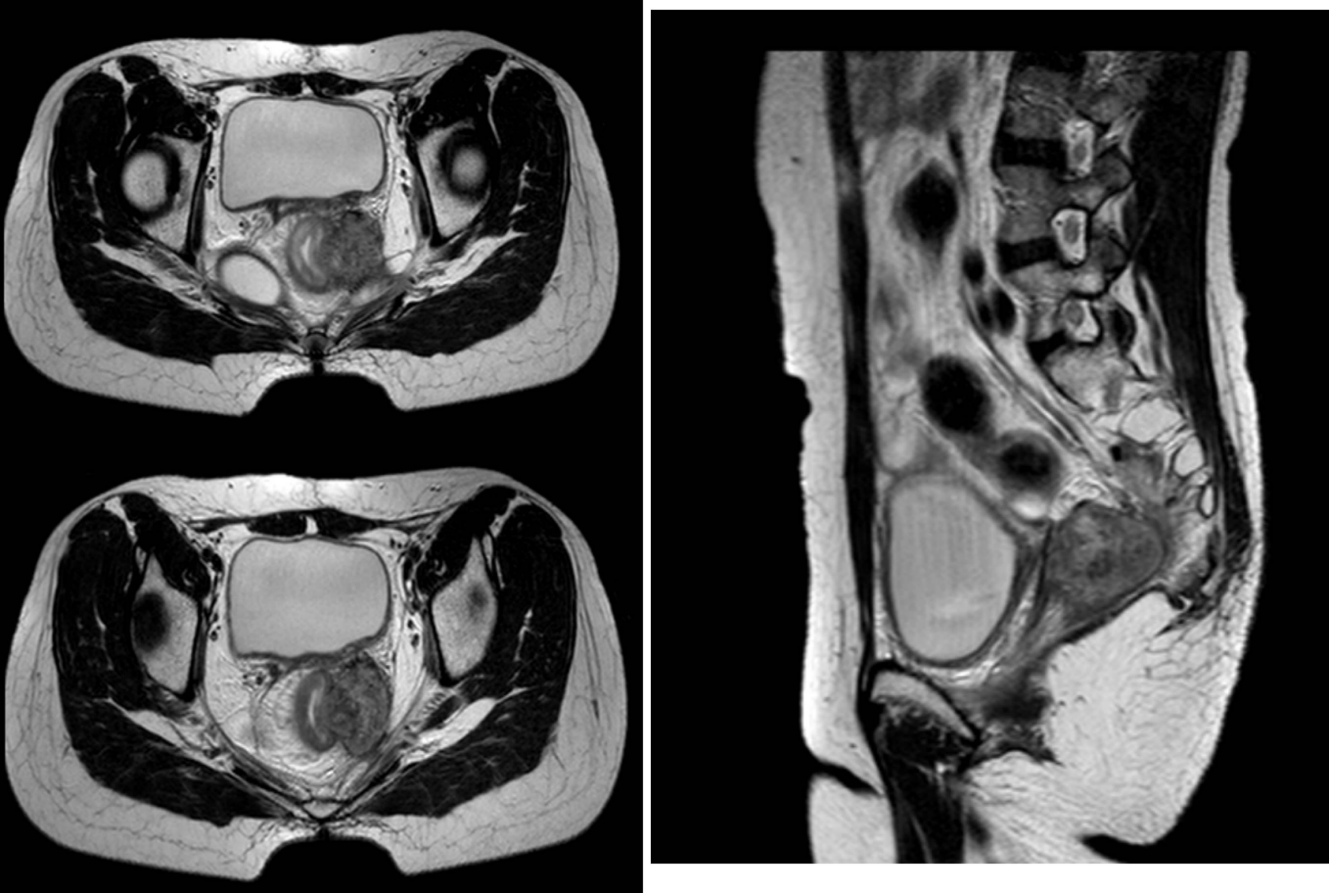
Pelvic MRI before reirradiation, recurrent tumors are shown in red circles.

**Figure 2:**
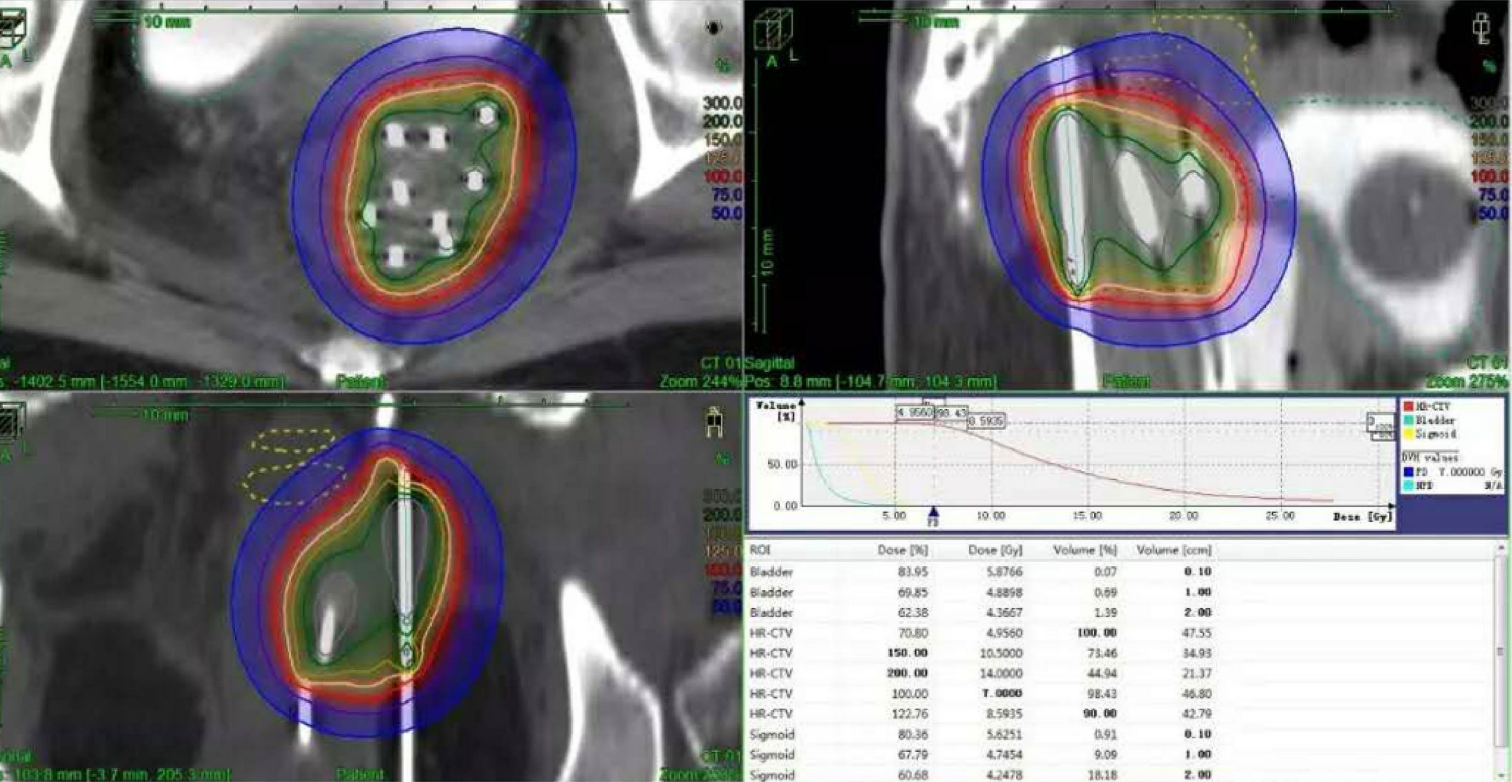
Distribution of needles and dose curves.

HDR-ISBT–based reirradiation was performed using three-dimensional (3D) planning in all patients. All the patients have pelvic MRI before brachytherapy. The Ir^192^ HDR brachytherapy machine, Oncentra brachytherapy planning system, and metal needle implantation are from Elekta, Sweden. The HDR ISBT was performed twice a week, 4–7 fractions in total, and 6–7 Gy for each fraction. Because of the large tumor volume and/or wide scope of invasion, ISBT was performed by freehand metal needle placement under CT guidance, resulting in excellent dose coverage. The patient underwent lumbar anesthesia in the operating room. After receiving anesthetic drugs, the patient was transferred to the large-caliber CT room dedicated to the Radiotherapy Department through a transfer bed. The anesthesiologist accompanied the patients all time to monitor vital signs. First, an intrauterine tandem was implanted in the uterine cavity. Then, some parallel and oblique metal needles (length of 18 cm, diameter of 1.3 mm, Elekta) were inserted into the tumor and lateral extensions at different degrees angled to the vagina at a width and depth of approximately 10 mm as initial implantation. The insertion position and number of these oblique needles were decided by T2-weighted MRI (**Figure 1**) and gynecological examination. Vaginal packing with gauze was used to push away the rectum and bladder. Finally, through multiple CT scans, we repeatedly adjusted the direction and depth of the needles until all the needles were evenly distributed within the tumor. In the treatment of all patients, including those with rectal and bladder involvement, the final position of the needle should be as far beyond the depth of invasion as possible, 0–1 cm, to obtain good target volume and dose distribution of the paddle in subsequent treatment plans (**Figure 2**).

### Dose-volume-histograms analysis

3D plans were reported in high-risk clinical target volume (HR-CTV), defined as the cervix and the involved parametrium, vagina, vulva, urethra, pelvic wall, rectum, and bladder. The following dose–volume parameters were calculated for the HR-CTV and OARs (bladder and rectum): the percentage of the CTV receiving 100% of the prescribed dose (V100) and the dose that covered 90%, 98%, and 100% of the target volume (D90, D98, and D100, respectively). We calculated the D2cc of the rectum and bladder. The total doses (EBRT and HDR-BT) were recalculated as the biologically equivalent doses to 2-Gy fractions (EQD2) using the following equation: EQD2 = nd (d + α/β/2 Gy + α/β), where n = the number of fractions, d = dose (Gy) per fraction (assuming α/β = 10 Gy for tumor control, and α/β = 3 Gy for late normal tissue damage). For primary BT, the dose delivered by the two-dimensional (2D) treatment plan was calculated at point A and the rectum and bladder reference points, based on International Commission on Radiation Units and Measurements (ICRU)89 report (18).

### Analysis of curative effect and toxicity after reirradiation

All time intervals were calculated from the final day of treatment. The tumor response was assessed 2 months after the reirradiation. The complete response (CR) was defined as the disappearance of the tumor, and the partial response (PR) was defined as a reduction of at least 30% in the sum of the maximum diameters of the tumor. Stable disease (SD) was defined as when none of the above conditions is applicable. Local control (LC) was defined as the length of time from the end of treatment to LR. The post-relapse survival (PRS) was calculated from the date of relapse diagnosis to the date of death for disease or the date of the last follow-up.

The bladder and rectum complications were scored using the Radiation Therapy Oncology Group Acute and Late Radiation Morbidity Scoring Criteria (19).

### Statistical analysis

PRS curves were derived from Kaplan–Meier estimates and compared using the log-rank test. The influence of potential prognostic factors on the risk of failure was assessed using a Cox model. *P*-values<0.05 were considered statistically significant.

## Results

### Patient characteristics

**Table 1** listed the detailed characteristics of patients. The median age of the patients at diagnosis was 50.5 years (range = 24–68 years). According to the International Federation of Gynecology and Obstetrics (FIGO) Cervical Cancer Staging 2009 standard (20), the clinical stage at the initial diagnosis was stages I to II in 17 patients and stages III to IV in six patients. Twenty-one patients had SCC, and two patients displayed non-SCC histologic finding. Seventeen (73.9%) patients had been treated with definitive radiotherapy and others had been treated with postoperative radiotherapy in primary radiotherapy. The median EQD2 calculated for Primary EBRT + BT was 92.0 Gy (range = 31.3–109.7 Gy). The median treatment-free interval (TFI) (between the end of primary radiotherapy and reirradiation) was 13 months (range = 3–93 months). Eleven patients (47.8%) were diagnosed with recurrence within 12 months of the initial radiotherapy. All patients had a performance status of 0 to 1, and the median (mean) tumor diameter at the time of recurrence diagnosis was 51.0(56.6) mm. the median (mean) HR-CTV at the time of reirradiation was 70.8 (82.9) cm^3^. Eleven patients had the number of tumor invasion organ including bladder, rectum, and pelvic wall greater than 1. The tumors of the other 12 patients did not invade the bladder, rectum, or pelvic wall.

**Table 1:** Baseline characteristics of involved patients (N = 23).

Characteristics	No. patients
Age (years)	
Median (range)	50.5 (24–68)
≤50	10 (43.5%)
>50	13 (56.5%)
FIGO stage*	
I–II	17 (73.9%)
III–IV	6 (26.1%)
Histologic finding	
SCC	21 (91.3%)
Non-SCC	2 (8.7%)
Prior radiotherapy	
Definitive	17 (73.9%)
Postoperative	6 (26.1%)
Primary EBRT + BT EQD2 (Gy)	
Median (range)	92.0 (31.3–109.7)
TFI(months)	
Median (range)	14 (3–93)
TFI ≤ 12	11 (47.8%)
TFI > 12	12 (52.2%)
Performance status	
0–1	23 (100%)
2–3	0 (0%)
Maximum tumor diameter (mm)	
Median (range)	51 (29–92)
≤50	11 (47.8%)
>50	12 (52.2%)
HR-CTV(cm^3^)	
Median (range)	70.8 (20.3–208.3)
≤80	12 (52.2%)
>80	11 (47.8%)
Tumor invasion organ number (bladder, rectum, and pelvic wall)	
0	12 (52.2%)
≥1	11 (47.8%)

*Clinical stage at the time of the initial diagnosis.

### Treatment outcome

There were 13 patients’ tumors that reached CR after radiotherapy, nine had PR, and one had SD (**Table 2**). The CR rate after reirradiation was 56.5% (13 of 23). The median follow-up time was 19 months (range = 2–59months). Six patients are alive with no evidence of disease (NED), from 33 to 59 months post-treatment (median = 48 months). One patient is alive with disease (AWD), receiving chemotherapy. Sixteen patients died. No patients were lost to follow-up. Eight patients received HDR-ISBT combined with EBRT reirradiation. Fifteen patients received HDR-ISBT alone (**Table 2**).

**Table 2:** Treatment outcomes of involved patients.

Clinical Outcomes		No. of Patients
Local control	CR	13 (56.5%)
	PR	9 (39.1%)
	SD	1 (4.3%)
Post-relapse survival (months)	Median (range)	19 (2–59)
Reirradiation modality	EBRT +ISBT	8 (34.8%)
	Only ISBT	15 (65.2%)
Reirradiation EBRT + BT EQD2 (Gy)	Median (range)	64.0 (31.3–95.1)
Reirradiation D90 (Gy)	Median (range)	37.8 (13.8–56.6)
Reirradiation D98 (Gy)	Median (range)	29.5 (10.9–47.1)
Reirradiation D100 (Gy)	Median (range)	23.7 (7.4–35.5)
Reirradiation V100 (%)	Median (range)	93.4 (78.4–98.4)
Primary RT + Reirradiation EQD2 (Gy)	Median (range)	152.4 (97.8–200.9)

The median EQD2 calculated for reirradiation was 64.0 Gy (range = 31.3–95.1 Gy), and the median cumulative EQD2 for primary treatment and reirradiation was 152.4 Gy (range = 97.8–200.9 Gy) (**Table 2**). The median D90, D98, and D100 were 37.8, 29.5, and 23.7 Gy in the re-radiotherapy planning, respectively. The median V100 was 93.4% (**Table 2**).

Tumor volume before reirradiation was found in six of the seven surviving patients was<80 cm^3^. The tumor invasion in 12 patients did not include the rectum, bladder, and pelvic wall, and the average follow-up time was 31.9 months (9–59 months). Five of them were NED, one was AWD, five were dead of oncologic disease (DOD), and one died due to cardiac sudden death. A total of 13 patients had CR in remission status after therapy, and the average follow-up time was 31.8 months. Among them, six were NED, one was AWD, three died due to LR or distant metastasis, two died due to complications of treatment, and one died due to cardiac sudden death. The treatment characteristics are detailed in (**Supplemental Table 1**).

**Figure 3:**
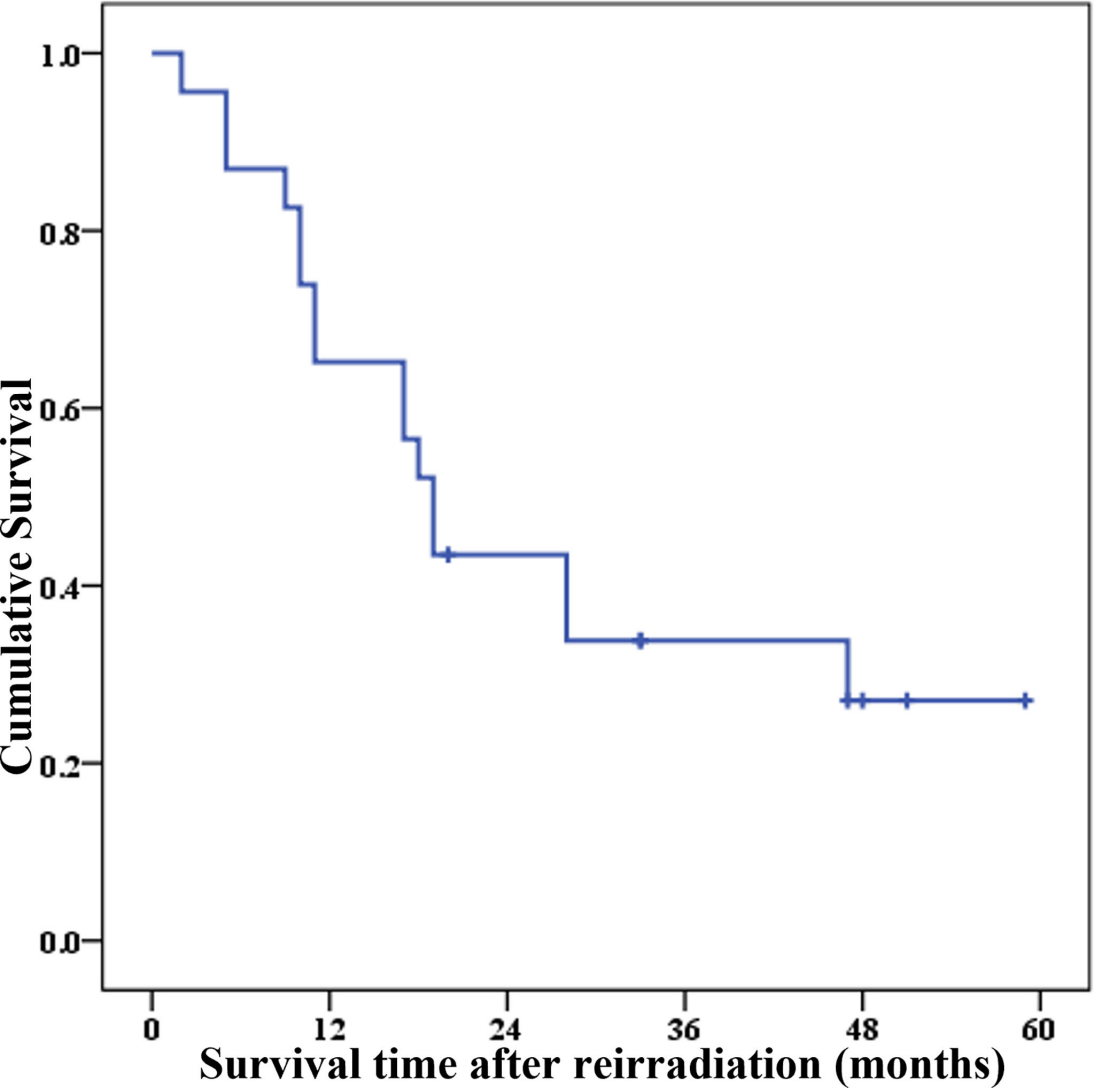
Survival curve of PRS after reirradiation in 23 patients with recurrent cervical cancer.

The 1-, 2-, 3-, and 4-year PRS rates were 65.2% (95% CI = 42.3%–80.8%), 43.5% (95% CI = 23.3%–62.1%), 33.8% (95% CI = 15.6%–53.1%), and 27.1% (95% CI = 10.2%–47.3%), respectively. The survival curve of PRS after re-radiotherapy is shown in **Figure 3**.

### Dosage delivered to OARs and toxicity

The median primary RT EQD2 D2cc of rectum and bladder was 73.5 Gy (range = 22.8–93.1 Gy) and 80.2 Gy (range = 22.8–106.0 Gy) (**Table 3**). The median reirradiation EQD2 D2cc of rectum and bladder was 39.5 Gy (range = 14.6–96.2 Gy) and 52.1 Gy (range = 29.1–114.2 Gy), respectively. The median cumulative EQD2 D2cc of rectum and bladder was 115.0 Gy (range = 84.4–189.3 Gy) and 130.5 Gy (range = 95.5–173.5 Gy), respectively. During follow-up, nine (39.1%) patients had experienced grade 3 or 4 late toxicities (**Table 3**). Grade ≥3 rectal toxicity occurred in three patients (13.0%). Grade ≥3 urinary toxicity occurred in five patients (21.7%). One patient (4.3%) had both grade ≥3 urinary and rectal toxicity. The dosimetric parameters of the OARs and radiation-related toxic effects are provided in **Supplemental Table 2**.

**Table 3:** Dosage delivered to OARs and toxicity.

Dosage and Toxicity			No. of Patients
Severe late toxicity	Patients with grade 3/4 toxicities		9 (39.1%)
Primary RT dose delivered to bladder EQD2 (Gy)	Median (range)		80.2 (22.8–106.0)
Reirradiation dose delivered to bladder EQD2 (Gy)	Median (range)		52.1 (29.1–114.2)
Cumulative dose delivered to the bladder after primary RT and reirradiation EQD2 (Gy)	Median (range)		130.5 (95.5–173.5)
Grade of late radiation damage to the bladder	<3		17 (73.9%)
	≥3		6(26.1%)
Primary RT dose delivered to rectum EQD2 (Gy)	Median (range)		73.5 (22.8–93.1)
Reirradiation dose delivered to rectum EQD2 (Gy)	Median (range)		39.5 (14.6–96.2)	
Cumulative dose delivered to the rectum after primary RT and reirradiation EQD2 (Gy)	Median (range)		115.0 (84.4–189.3)	
Grade of late radiation damage to the rectum	<3		19 (82.6%)	
	≥3		4 (17.4%)	

### Predictors of survival after reirradiation

**Figure 4:**
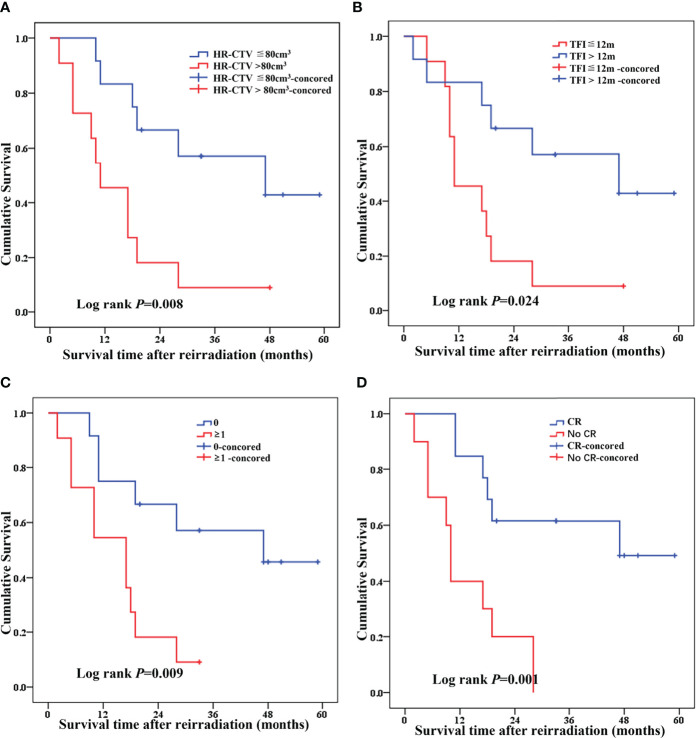
Cox model analysis of independent predictors of treatment outcome in recurrence patients receiving re-radiotherapy [**(A)** HR-CTV, **(B)** TFI, **(C)** tumor invasion organ number (bladder, rectum, and pelvic wall), and **(D)** local control].

We performed Cox model analysis to identify independent predictors of treatment outcome in patients receiving re-radiotherapy (**Table 4** and **Figures 4A–D**). Cox model analysis identified four independent prognostic factors that predicted good outcomes: Tumor volume (*P* = 0.008, **Table 4** and **Figure 4A**), TFI (*P* = 0.024, **Table 4** and **Figure 4B**), tumor invasion organ number (*P* = 0.009, **Table 4** and **Figure 4C**), and LC (*P* = 0.001, **Table 4** and **Figure 4D**).

**Table 4:** Univariate analysis of survival after reirradiation.

Characteristics		N (%)	Median OS	Log-Rank *P*
Tumor volume	≤80 cm^3^	12 (50%)	47	0.008
	>80 cm^3^	11 (90.9%)	11	
Treatment-free interval	≤12 m	11 (91.9%)	11	0.024
	>12 m	12 (50%)	47	
Tumor diameter	≤5cm	11 (54.5%)	47	0.197
	>5 cm	12 (83.3%)	17	
Tumor invasion organ number (bladder, rectum, and pelvic wall)	0	12 (50%)	47	0.009
	≥1	11 (90.9%)	17	
Reirradiation dose	≤64 Gy	12 (83.3%)	19	0.545
	>64GY	11 (54.5%)	17	
Local control	CR	13 (46.2%)	47	0.001
	No CR	10 (100%)	10	
FIGO stage	I–II	17 (70.6%)	18	0.678
	III–IV	6 (66.7%)	19	

The statistically significant factors, including tumor volume, TFI, tumor invasion organ number, and LC, were included in the multivariate Cox risk ratio model. The results showed that, after adjusting for TFI, tumor invasion organ number and LC were the independent factors for patient survival. The risk of death in patients with TFI≦12 months was 4.694 times higher than patients with >12 months (HR = 4.694, 95% CI = 1.445–15.247, **Table 4** and **Figure 4B**). The risk of death in patients with tumor invasion organ number≥1 was 3.708 times higher than patients with = 0 (HR = 3.708, 95% CI = 1.048–13.116, **Table 4** and **Figure 4C**). The risk of death in patients with no-CR was 3.617 times higher than patients with CR (HR = 3.617, 95% CI =1.155–11.330, **Table 4** and **Figure 4D**).

## Discussion

For patients with recurrent cervical cancer in the original irradiation field, the possible efficacy and potential toxicity should be considered during treatment. Previous studies have shown that the 5-year OS of patients with recurrent cervical cancer after radiotherapy is only 1% if they do not receive any treatments (21). ISBT can generate favorable LC in recurrent tumors that cannot be adequately covered by ICBT (22). Recent reports have shown that HDR-ISBT re-radiotherapy has good disease control and acceptable complications (11–15). Therefore, ISBT had been applied to patients with recurrent cervical cancer in our department. In our study, the 1-, 2-, 3-, and 4-year PRS rates were 65.2%, 43.5%, 33.8%, and 27.1%, respectively, and the median dose of EQD2 for reirradiation was 64 Gy. The CR rate after reirradiation was 56.5%. Nine (39.1%) patients had experienced grade 3 or 4 late toxicities. Grade ≥3 rectal toxicity occurred in three patients (13.0%). Grade ≥3 urinary toxicity occurred in five patients (21.7%). One patient (4.3%) had both grade ≥3 urinary and rectal toxicity. The doses used in our study were comparable to those in the current literature (11–14). Some of the toxicity symptoms are associated with a large recurrence of the rectum, bladder, urethra, etc. Because of its wide recurrence range and wide irradiation range, the damage of adjacent normal tissues is serious. In addition, some patients failed to achieve CR after treatment, and the corresponding toxic symptoms may be related to tumor control or HDR dose, which is difficult to identify. Therefore, we reported all the toxic symptoms of the patients and counted them as the toxic reactions caused by the treatment, which resulted in more toxic reactions in our study than in other reports. It is difficult to compare these results because the study cohorts were heterogeneous in terms of histopathology, tumor size, location of recurrence, treatment method, and RT schedules.

The main difficulty with re-radiotherapy is that the surrounding normal tissue is near the tumor but cannot receive high doses of radiation. A recent study showed that ISBT could be administered by injecting biodegradable hydrogels to increase the distance between the target and adjacent normal tissue, thereby reducing normal tissue exposure and therapeutic toxicity (23).

In our retrospective study, we found that, for patients with LR of gynecological tumors after radiotherapy, the use of HDR ISBT can cure the patient with acceptable side effects. In particular, patients with small local tumor volume and uninvaded pelvic wall, rectum, and bladder have a better chance of cure. If the local tumor reaches CR after treatment, then a good prognosis is suggested. However, there are no published guidelines for the dose and normal tissue limits of relapsing pelvic radiotherapy for cervical cancer. Current literature reports are all small sample studies.

Liu et al. (24) reported that freehand ISBT resulted in D90 HR-CTV of 87 Gy or greater in 85.7% of patients; however, D90 HR-CTV of ICBT was 87 Gy or greater only in 6.7% of the cases, which are bulky tumors and/or parametrial extension (tumor size>5 cm) after external beam radiotherapy. In our study, the average CTV was 88.5 cm^3^ (range: 26.9–208.3 cm^3^), and the median percentage volume of 100% prescription dose was 93.1%. This result suggests that freehand ISBT has better dose–volume histogram parameters for large volume tumors than conventional ICBT.

Permanent interstitial brachytherapy (PIB) is also a viable and potentially durable treatment modality that can be used to treat recurrent pelvic malignancies in the field of previous irradiation. Feddock et al. (25) reported on PIB for the treatment of recurrent pelvic malignancies with ^131^Cs and ^198^Au as an isotope of PIB. The 2-year success rate for reirradiation using PIB was 67.3%. Grade 3 to 4 toxicities were observed in eight patients (16.7%). However, the limitation in this series is the fact that majority of recurrences in this series were small volume, with an area of 6 cm^3^. In our study, the average CTV was 88.5 cm^3^.

To gain some insight from patients who benefited from re-radiotherapy, we performed a retrospective analysis of disease-free patients after treatment. Only one of the six NED patients involved the rectum and pelvic wall, and all of the remaining patients excluded the bladder, rectum, and pelvic wall. Additional external radiotherapy was used in only one patient with rectum and pelvic wall involvement, whereas brachytherapy was used in all of the patients. The TFI was 13.8 months (7–23 months), the mean tumor volume was 77.5cm^3^ (39.7–136.9cm^3^), the mean EQD2 of reirradiation was 69.8 Gy (51.3–95.1 Gy), the mean EQD2 calculated for primary RT and reirradiation was 157.3 Gy (143.3–184.4 Gy), and the mean follow-up time was 32 months (20–46 months). One patient underwent transverse colostomy prior to reirradiation for rectal invasion. Hence, the rectal dose of this patient was not calculated. The average bladder and rectum doses of re-radiotherapy were 52.5 Gy (40.9–66.3 Gy) and 41.5 Gy (35.9–47.6 Gy), respectively. The median EQD2 calculated for primary RT and reirradiation of bladder and rectum were 136.0 Gy (90.5–148.8 Gy) and 120.0Gy (101.8–126.0 Gy), respectively. Two patients had a grade ≥3 urologic radiation response (two patients had a vaginal bladder fistula at 10 and 23 months, respectively), and one patient had a grade ≥3 gastrointestinal radiation response (1 patient had a colorectal fistula at 26 months after radiation) (**Tables 1** and **2**, supporting information). It has been reported that the cumulative dose of EQD2 in the bladder and rectum below 100 Gy is safe for HDR-BT reradiation (11). In our study, we found that, if the tumor could disappear, then the therapeutic toxicity caused by the cumulative dose of 136 and 120 Gy in the bladder and rectum was acceptable. If LC is poor, then serious complications may not occur before death.

Considering that HDR-ISBT reirradiation can lead to serious complications, selecting the individuals who would benefit from treatment is critical. Mabuchi et al. (14) suggested that tumor-free survival (>6 months), tumor diameter (<40 mm), and the initial FIGO staging (I–II) prognosis were good. The more the combined risk factors, the worse the prognosis and the lower the treatment benefit. In the study of Weitmann et al. (26), recurrence time > 2 years, initial tumor diameter ≤4cm, initial volume <15 cm^3^, no pelvic lateral wall invasion, volume before BT< 7.5 cm^3^, and the prescribed dose > 64 Gy were positive predictors. Mahantshetty et al. (13) suggested that patients who received >40-Gy EQD2 reirradiation dose and the interval between two radiotherapy sessions>25 months had a better prognosis. However, because of the sample size, these data were not statistically significant. Zolciak-Siwinska et al. (11) showed that the interval between initial RT and reirradiation ≤12 months and the tumor diameter > 3 cm had a poor prognosis.

Our results confirmed that tumor volume ≤ 80 cm^3^ before reirradiation, TFI >12 months, local tumor reaching CR after reirradiation, and no tissue invasion of pelvic wall, rectum, and bladder were the factors for a better prognosis. The dose level did not affect PRS. This observation may be due to the small size and heterogeneity of the study group or due to our higher prescribing dose. For patients with larger tumors and wider areas of invasion, we give higher doses, because we want to increase the local dose of the tumor to improve the LC rate, and the limitation of normal tissue is not too strict. Therefore, the prognosis of patients with the re-radiotherapy dose of > 64 Gy was worse. Further large-scale prospective clinical studies are needed to select suitable patients for HDR-ISBT reirradiation.

## Conclusions

To sum up, our findings revealed that HDR-BT reirradiation is clinically feasible in patients with recurrent cervical cancer after radiotherapy, even if the local tumor invasion is large, there is a good chance of survival and an acceptable risk of complications. We suggest that the cumulative dose of EQD2 in the bladder and rectum can be relaxed to 136 and 120 Gy when ISBT re-radiotherapy is performed for recurrent cervical cancer with a chance of radical cure.

## Data availability statement

The original contributions presented in the study are included in the article/**supplementary material**. Further inquiries can be directed to the corresponding author.

## Ethics statement

The studies involving human participants were reviewed and approved by the ethics committee of the Second Hospital of Jilin University. The patients/participants provided their written informed consent to participate in this study. Written informed consent was obtained from the individual(s) for the publication of any potentially identifiable images or data included in this article.

## Author contributions

XR conceived and designed the experiments, performed the experiments, analyzed the data, prepared figures and/or tables, authored or reviewed drafts of the paper, and approved the final draft. YF performed the experiments, analyzed the data, prepared figures and/or tables, authored or reviewed drafts of the paper, and approved the final draft. ZL analyzed the data, authored or reviewed drafts of the paper, and approved the final draft. XL conceived and designed the experiments, performed the experiments, and approved the final draft. LQ performed the experiments, prepared figures and/or tables, authored or reviewed drafts of the paper, and approved the final draft. YL performed the experiments, analyzed the data, authored or reviewed drafts of the paper, and approved the final draft. HL performed the experiments, authored or reviewed drafts of the paper, and approved the final draft. YB performed the experiments, authored or reviewed drafts of the paper, and approved the final draft. TW conceived and designed the experiments, performed the experiments, analyzed the data, authored or reviewed drafts of the paper, and approved the final draft. All authors contributed to the article and approved the submitted version.

## Funding

This work was funded by the National Key Clinical Specialty Capacity Building Project (Application of Non-coplanar 3D Printing and Intertissue Interpolation Technology in Improving the Diagnosis and Treatment Ability of Recurrent and Refractory Cervical Cancer), Jilin Province Financial and Health Project (Research on the Differential Expression of Cyclic RNA in Cervical Cancer HeLa Cells and the Mechanism of Radiation Resistance under Radiation Induction), Jilin Provincial Department of Finance (Consortium of Medical Consortium for Diagnosis and Treatment of Difficult Women’s Tumors and Precision Radiotherapy Training Base Construction Project), and Jilin Province Medical and Health Talent Special Project (2019SCZT010).

## Conflict of interest

The authors declare that the research was conducted in the absence of any commercial or financial relationships that could be construed as a potential conflict of interest.

## Publisher’s note

All claims expressed in this article are solely those of the authors and do not necessarily represent those of their affiliated organizations, or those of the publisher, the editors and the reviewers. Any product that may be evaluated in this article, or claim that may be made by its manufacturer, is not guaranteed or endorsed by the publisher.

## References

[1] Sung H Ferlay J Siegel RL Laversanne M Soerjomataram I Jemal A. Global cancer statistics 2020: Globocan estimates of incidence and mortality worldwide for 36 cancers in 185 countries. CA: Cancer J Clin (2021) 71(3):209–49. doi: 10.3322/caac.21660 33538338

[2] Quinn MA Benedet JL Odicino F Maisonneuve P Beller U Creasman WT. Carcinoma of the cervix uteri. figo 26th annual report on the results of treatment in gynecological cancer. Int J Gynaecol Obstetrics (2006) 95 Suppl 1:S43–103. doi: 10.1016/s0020-7292(06)60030-1 17161167

[3] Perez CA Grigsby PW Camel HM Galakatos AE Mutch D Lockett MA. Irradiation alone or combined with surgery in stage ib, iia, and iib carcinoma of uterine cervix: Update of a nonrandomized comparison. Int J Radiat Oncol Biol Phys (1995) 31(4):703–16. doi: 10.1016/0360-3016(94)00523-0 7860381

[4] Hong JH Tsai CS Lai CH Chang TC Wang CC Chou HH. Recurrent squamous cell carcinoma of cervix after definitive radiotherapy. Int J Radiat Oncol Biol Phys (2004) 60(1):249–57. doi: 10.1016/j.ijrobp.2004.02.044 15337563

[5] Eralp Y Saip P Sakar B Kucucuk S Aydiner A Dincer M. Prognostic factors and survival in patients with metastatic or recurrent carcinoma of the uterine cervix. Int J Gynecol Cancer (2003) 13(4):497–504. doi: 10.1046/j.1525-1438.2003.13325.x 12911727

[6] Monk BJ Tewari KS. Evidence-based therapy for recurrent cervical cancer. J Clin Oncol (2014) 32(25):2687–90. doi: 10.1200/jco.2014.56.8733 25071120

[7] Mabuchi S Matsumoto Y Komura N Sawada M Tanaka M Yokoi E. The efficacy of surgical treatment of recurrent or persistent cervical cancer that develops in a previously irradiated field: A monoinstitutional experience. Int J Clin Oncol (2017) 22(5):927–36. doi: 10.1007/s10147-017-1134-x 28551815

[8] Berek JS Howe C Lagasse LD Hacker NF. Pelvic exenteration for recurrent gynecologic malignancy: Survival and morbidity analysis of the 45-year experience at ucla. Gynecol Oncol (2005) 99(1):153–9. doi: 10.1016/j.ygyno.2005.05.034 16054678

[9] Chiva LM Lapuente F González-Cortijo L González-Martín A Rojo A García JF. Surgical treatment of recurrent cervical cancer: State of the art and new achievements. Gynecol Oncol (2008) 110(3 Suppl 2):S60–6. doi: 10.1016/j.ygyno.2008.05.024 18639923

[10] Boers A Arts HJ Klip H Nijhuis ER Pras E Hollema H. Radical surgery in patients with residual disease after (Chemo)Radiation for cervical cancer. Int J Gynecol Cancer (2014) 24(7):1276–85. doi: 10.1097/igc.0000000000000171 24987914

[11] Zolciak-Siwinska A Bijok M Jonska-Gmyrek J Kawczynska M Kepka L Bujko K. Hdr brachytherapy for the reirradiation of cervical and vaginal cancer: Analysis of efficacy and dosage delivered to organs at risk. Gynecol Oncol (2014) 132(1):93–7. doi: 10.1016/j.ygyno.2013.10.018 24161366

[12] Martínez-Monge R Cambeiro M Rodríguez-Ruiz ME Olarte A Ramos LI Villafranca E. Phase ii trial of image-based high-Dose-Rate interstitial brachytherapy for previously irradiated gynecologic cancer. Brachytherapy (2014) 13(3):219–24. doi: 10.1016/j.brachy.2014.01.008 24559794

[13] Mahantshetty U Kalyani N Engineer R Chopra S Jamema S Ghadi Y. Reirradiation using high-Dose-Rate brachytherapy in recurrent carcinoma of uterine cervix. Brachytherapy (2014) 13(6):548–53. doi: 10.1016/j.brachy.2014.06.005 25085457

[14] Mabuchi S Takahashi R Isohashi F Yokoi T Okazawa M Sasano T. Reirradiation using high-Dose-Rate interstitial brachytherapy for locally recurrent cervical cancer: A single institutional experience. Int J Gynecol Cancer (2014) 24(1):141–8. doi: 10.1097/igc.0000000000000028 24362719

[15] Badakh DK Grover AH. Reirradiation with high-Dose-Rate remote afterloading brachytherapy implant in patients with locally recurrent or residual cervical carcinoma. J Cancer Res Ther (2009) 5(1):24–30. doi: 10.4103/0973-1482.48766 19293485

[16] Demanes DJ Rodriguez RR Bendre DD Ewing TL. High dose rate transperineal interstitial brachytherapy for cervical cancer: High pelvic control and low complication rates. Int J Radiat Oncol Biol Phys (1999) 45(1):105–12. doi: 10.1016/s0360-3016(99)00124-8 10477013

[17] Georg P Lang S Dimopoulos JC Dörr W Sturdza AE Berger D. Dose-volume histogram parameters and late side effects in magnetic resonance image-guided adaptive cervical cancer brachytherapy. Int J Radiat Oncol Biol Phys (2011) 79(2):356–62. doi: 10.1016/j.ijrobp.2009.11.002 20385450

[18] . doi: 10.1093/jicru/ndw042

[19] Cox JD Stetz J Pajak TF. Toxicity criteria of the radiation therapy oncology group (Rtog) and the European organization for research and treatment of cancer (Eortc). Int J Radiat Oncol Biol Phys (1995) 31(5):1341–6. doi: 10.1016/0360-3016(95)00060-c 7713792

[20] Pecorelli S Zigliani L Odicino F. Revised figo staging for carcinoma of the cervix. Int J Gynaecol Obstetrics (2009) 105(2):107–8. doi: 10.1016/j.ijgo.2009.02.009 19342051

[21] Sommers GM Grigsby PW Perez CA Camel HM Kao MS Galakatos AE. Outcome of recurrent cervical carcinoma following definitive irradiation. Gynecol Oncol (1989) 35(2):150–5. doi: 10.1016/0090-8258(89)90033-4 2807004

[22] Murakami N Kobayashi K Shima S Tsuchida K Kashihara T Tselis N. A hybrid technique of intracavitary and interstitial brachytherapy for locally advanced cervical cancer: Initial outcomes of a single-institute experience. BMC Cancer (2019) 19(1):221. doi: 10.1186/s12885-019-5430-x 30866877 PMC6417107

[23] Elledge CR LaVigne AW Bhatia RK Viswanathan AN. Aiming for 100% local control in locally advanced cervical cancer: The role of complex brachytherapy applicators and intraprocedural imaging. Semin Radiat Oncol (2020) 30(4):300–10. doi: 10.1016/j.semradonc.2020.05.002 PMC7875154 32828386

[24] Liu ZS Guo J Zhao YZ Lin X Zhang BY Zhang C. Computed tomography-guided interstitial brachytherapy for locally advanced cervical cancer: Introduction of the technique and a comparison of dosimetry with conventional intracavitary brachytherapy. Int J Gynecol Cancer (2017) 27(4):768–75. doi: 10.1097/igc.0000000000000929 PMC5405778 28267131

[25] Feddock J Cheek D Steber C Edwards J Slone S Luo W. Reirradiation using permanent interstitial brachytherapy: A potentially durable technique for salvaging recurrent pelvic malignancies. Int J Radiat Oncol Biol Phys (2017) 99(5):1225–33. doi: 10.1016/j.ijrobp.2017.08.027 29029888

[26] Weitmann HD Knocke TH Waldhäusl C Pötter R. Ultrasound-guided interstitial brachytherapy in the treatment of advanced vaginal recurrences from cervical and endometrial carcinoma. Strahlenther Und Onkol: Organ Der Deutschen Rontgengesellschaft (2006) 182(2):86–95. doi: 10.1007/s00066-006-1420-4 16447015

