# Supplementary material for: Image-guided interstitial brachytherapy for recurrent cervical cancer after radiotherapy: A single institution experience
Source: Front Oncol. 2022 Jul 19;12:943703. doi: 10.3389/fonc.2022.943703 (PMC9344972; doi:10.3389/fonc.2022.943703)
Supplement: Supplementary file 1 [file Table_1.docx]

**Table 1. Patient Characteristics and Treatment Outcomes.**

| **Patient number** | **Surgery as primary treatment Yes/No** | **Interval between RT and reirradiation**  **(months)** | **Tumor location** | **Primary EBRT+BT dose(Gy)/fractions number/technique** | **Primary EBRT+BT EQD2(Gy)** | **Re-irradiation EBRT+BT dose(Gy)/fractions number** | **Re-irradiation EBRT+BT EQD2(Gy)** | **Re-irradiation D90** | **Tumor volume（cm^3^）** | **Primary RT+re-irradiation EQD2(Gy)** | **Remission status after therapy** | **Duration of follow-up in months** |
| --- | --- | --- | --- | --- | --- | --- | --- | --- | --- | --- | --- | --- |
| **1** | **No** | **7** | **C, P** | **50/25+30/5(ICBT)** | **90.0** | **35/5** | **62.4** | **41.0** | **136.9** | **152.4** | **CR** | **48 NED** |
| **2** | **No** | **6** | **C, P** | **50.4/28+30/5（ISBT）** | **92.5** | **24/4** | **31.3** | **23.6** | **145.1** | **123.8** | **PR** | **9 death** |
| **3** | **No** | **14** | **C, V** | **50.4/28+30/5（ISBT）** | **92.0** | **30/5** | **51.3** | **37.8** | **39.7** | **143.3** | **CR** | **47 death** |
| **4** | **No** | **15** | **P, W** | **50.4/28+36/6（ISBT）** | **104.4** | **36/6** | **51.6** | **37.9** | **95.1** | **156.0** | **PR** | **10 death** |
| **5** | **No** | **8** | **V,C,P** | **45/25+30/5 (ICBT)** | **84.3** | **30/5** | **38.6** | **29.2** | **88.5** | **122.8** | **PR** | **28 death** |
| **6** | **No** | **3** | **C,V** | **50.4/28+36/6（ISBT）** | **96.8** | **42/6** | **67.5** | **45.8** | **112.5** | **164.2** | **CR** | **11 death** |
| **7** | **No** | **6** | **C,V,P** | **50/25+30/5+10/2(ICBT)** | **109.7** | **31/5** | **50.0** | **35.1** | **37.3** | **159.7** | **CR** | **19 death** |
| **8** | **No** | **58** | **A,V** | **50/25+28/4+12/2(ICBT)** | **106.0** | **50/25+30/6** | **94.9** | **34.1** | **26.9** | **200.9** | **CR** | **51 NED** |
| **9** | **No** | **20** | **C,P** | **45/25+30/5（ISBT）** | **100.1** | **49/7** | **84.3** | **56.1** | **65.8** | **184.4** | **CR** | **33 NED** |
| **10** | **Yes** | **23** | **V,P,W,R** | **50.4/28** | **49.6** | **30/15+35/5** | **95.1** | **42.3** | **70.8** | **144.7** | **CR** | **33 NED** |
| **11** | **Yes** | **13** | **P,W** | **45/25+10/2 (ICBT)** | **66.8** | **30/15+28/4** | **77.8** | **31.9** | **41.7** | **144.6** | **PR** | **10 death** |
| **12** | **No** | **6** | **C,P,R** | **50/25+30/5 (ICBT)** | **90.0** | **30/5** | **47.9** | **34.1** | **68.5** | **137.9** | **CR** | **18 death** |
| **13** | **Yes** | **93** | **V,B** | **50/25+10/2 (ICBT)** | **62.5** | **30/15+27/4** | **64.0** | **24.9** | **51.7** | **126.5** | **PR** | **28 death** |
| **14** | **Yes** | **93** | **V,P** | **25/5 (ICBT)** | **31.3** | **50.4/28+40/6** | **91.9** | **42.3** | **114.2** | **123.2** | **SD** | **17 death** |
| **15** | **No** | **18** | **C,P,B** | **50.4/28+30/5（ISBT）** | **92.6** | **40/20+21/3** | **71.9** | **22.1** | **89.0** | **164.6** | **PR** | **5 death** |
| **16** | **No** | **6** | **C,V,P,W** | **60/25** | **62.0** | **30/15+32/5** | **76.9** | **33.5** | **144.5** | **138.9** | **CR** | **17 death** |
| **17** | **No** | **3** | **C,P,W,B,R** | **50/25** | **50.0** | **40/20+13/2** | **60.3** | **13.8** | **208.3** | **110.3** | **PR** | **5 death** |
| **18** | **Yes** | **22** | **C,B** | **50/25+10/2 (ICBT)** | **62.5** | **31/5** | **35.3** | **27.2** | **88.2** | **97.8** | **PR** | **19 death** |
| **19** | **No** | **21** | **C,V,P,W** | **50/25+30/5 (ICBT)** | **90.0** | **49/7** | **76.8** | **56.6** | **82.7** | **166.8** | **PR** | **2 death** |
| **20** | **Yes** | **8** | **V,P** | **20/4(ICBT)+45/25+20/4(ICBT)** | **94.3** | **43/7** | **58.3** | **43.2** | **61.2** | **152.6** | **CR** | **59 NED** |
| **21** | **No** | **11** | **C,V** | **45/25+36/6（ISBT）** | **99.1** | **44/7** | **67.4** | **47.9** | **90.6** | **166.5** | **CR** | **47 NED** |
| **22** | **No** | **22** | **P** | **45/25+32/5（ISBT）** | **102.9** | **35/5** | **65.3** | **42.4** | **20.3** | **168.2** | **CR** | **20 AWD** |
| **23** | **No** | **11** | **C,P** | **50.4/28+32/5（ISBT）** | **96.0** | **35/5** | **62.1** | **40.9** | **46.3** | **158.1** | **CR** | **11 death** |

***Note***: A: vulva, AWD: alive with disease, B: bladder, BT: brachytherapy, C: cervix, CR: complete remission, D90: 90% target BT dose, EBRT: external-beam therapy, EQD2: Equivalent dose for the tumor, assuming α/β = 10, NED: no evidence of disease; ICBT: intracavitary brachytherapy, ISBT: interstitial brachytherapy, P: parametrium, PR: partial remission, R: rectum, V: vagina, W: pelvic wall.

**Table 2 Cumulative dosage delivered to OAR (Gy) after primary radiotherapy and reirradiation, and late radiation damage analysis based on the RTOG score Criteria. Equivalent dose (EQD2) delivered to OAR, assuming α/β = 3.**

| **Patient number** | **Primary RT dose delivered to bladder EQD2(Gy)** | **Reirradiation dose delivered to bladder EQD2(Gy)** | **Cumulative dose delivered to the bladder after primary RT and reirradiation EQD2(Gy)** | **Grade of late radiation damage to the bladder** | **Primary RT dose delivered to rectum EQD2(Gy)** | **Reirradiation dose delivered to rectum EQD2(Gy)** | **Cumulative dose delivered to the rectum after primary RT and reirradiation EQD2(Gy)** | **Grade of late radiation damage to the rectum** |
| --- | --- | --- | --- | --- | --- | --- | --- | --- |
| **1** | **80.3** | **50.3** | **130.5** | **2** | **80.3** | **45.1** | **125.4** | **2** |
| **2** | **88.7** | **35.6** | **124.3** | **2** | **79.5** | **26.6** | **106.1** | **0** |
| **3** | **83.6** | **41.7** | **125.2** | **4** | **88.1** | **37.4** | **125.5** | **0** |
| **4** | **94.2** | **42.0** | **136.2** | **1** | **76.7** | **34.6** | **111.3** | **4** |
| **5** | **73.5** | **44.2** | **117.6** | **0** | **73.5** | **34.2** | **107.7** | **0** |
| **6** | **98.2** | **75.3** | **173.5** | **3** | **64.6** | **24.5** | **89.1** | **0** |
| **7** | **106.0** | **30.0** | **136.0** | **4** | **89.3** | **21.4** | **110.7** | **1** |
| **8** | **93.1** | **78.3** | **171.4** | **4** | **93.1** | **96.2** | **189.3** | **1** |
| **9** | **83.1** | **65.7** | **148.8** | **0** | **65.9** | **35.9** | **101.8** | **0** |
| **10** | **49.6** | **70.9** | **120.5** | **1** | **49.6** | **-** | **-** | **0** |
| **11** | **52.3** | **52.1** | **104.4** | **0** | **52.3** | **52.1** | **104.4** | **4** |
| **12** | **80.2** | **31.1** | **111.3** | **1** | **80.2** | **30.9** | **111.1** | **1** |
| **13** | **59.1** | **67.3** | **126.4** | **2** | **59.1** | **67.8** | **126.9** | **1** |
| **14** | **22.8** | **114.2** | **136.9** | **2** | **22.8** | **96.0** | **118.8** | **2** |
| **15** | **83.7** | **80.8** | **164.5** | **1** | **75.2** | **65.5** | **140.8** | **2** |
| **16** | **64.8** | **81.4** | **146.2** | **1** | **64.8** | **63.9** | **128.7** | **0** |
| **17** | **50.0** | **64.2** | **114.2** | **2** | **50.0** | **89.7** | **139.7** | **0** |
| **18** | **59.1** | **43.3** | **102.4** | **1** | **59.1** | **37.0** | **96.1** | **3** |
| **19** | **80.3** | **62.7** | **142.9** | **1** | **80.3** | **49.8** | **130.1** | **1** |
| **20** | **79.6** | **66.3** | **145.9** | **4** | **79.6** | **41.6** | **121.2** | **4** |
| **21** | **89.0** | **50.4** | **139.3** | **1** | **78.4** | **47.6** | **126.0** | **2** |
| **22** | **75.9** | **34.6** | **110.5** | **2** | **69.8** | **14.6** | **84.4** | **1** |
| **23** | **66.4** | **29.1** | **95.5** | **4** | **62.2** | **34.3** | **96.4** | **1** |
